# Is Reconsolidation a General Property of Memory?

**DOI:** 10.3389/fnhum.2021.643106

**Published:** 2021-02-26

**Authors:** Gayoung Kim, Minjae Kwon, Wonjun Kang, Sue-Hyun Lee

**Affiliations:** ^1^Department of Bio and Brain Engineering, College of Engineering, Korea Advanced Institute of Science and Technology, Daejeon, South Korea; ^2^Program of Brain and Cognitive Engineering, College of Engineering, Korea Advanced Institute of Science and Technology, Daejeon, South Korea

**Keywords:** reconsolidation, memory retrieval, declarative memory, cortical circuit, memory age

## Abstract

Memory reconsolidation holds great hope for memory modification approaches and clinical treatments of mental disorders associated with maladaptive memories. However, it remains controversial as to whether reconsolidation is a general property of all types of memory. Especially, discrepancies have been reported in research focusing on whether declarative memory undergoes reconsolidation, and whether old memories can be reorganized after retrieval. Here, we discuss how these inconsistent results can be reconciled and what information we need to uncover for the general use of reconsolidation.

Reconsolidation theory has attracted the attention of many researchers because it suggests the possibility that memory can be modified depending on the experience or manipulation given after retrieval within a limited labile time window (Nader et al., 2000; Tronson and Taylor, 2007; Lee et al., 2008; Lee, 2009; Nader and Hardt, 2009; Schiller and Phelps, 2011; Elsey et al., 2018). Especially this theory provides great hope for generating potential therapeutic approaches for treating mental disorders caused by traumatic memories such as post-traumatic stress disorder (PTSD) (Schwabe et al., 2014; Lee et al., 2017).

Since the idea of reconsolidation was initially suggested (Misanin et al., 1968; Schneider and Sherman, 1968), work by Nader et al. (2000) has accelerated reconsolidation research in the field of memory. They showed the impairment of long-term fear memory in rats through an injection of a protein synthesis inhibitor into the amygdala immediately after memory retrieval (Nader et al., 2000). Based on these findings in rodent models, whether such reconsolidation also occurs in human subjects were tested and demonstrated (Schiller and Phelps, 2011; Elsey et al., 2018). Because injection of protein synthesis inhibitors cannot be easily applied to humans, behavioral interference or pharmacological manipulation using propranolol instead of a protein synthesis inhibitor injection was used. Walker et al. (2003) showed that motor memory acquired through training involving a finger-tapping sequence can be disrupted by learning a new finger-tapping sequence if participants retrieved the first learned sequence prior to learning the second sequence. Reconsolidation of Pavlovian fear memory as found in rodents has also been observed in humans. Interfering with reconsolidation by extinction training in the labile window after retrieval impaired consolidated fear memory (Schiller et al., 2010; Oyarzún et al., 2012). Moreover, the reconsolidation interference successfully reduced expression of fear among phobia patients by applying propranolol or extinction training after reactivation (Soeter and Kindt, 2015; Björkstrand et al., 2016), and the effects were sustained over 6 months (Björkstrand et al., 2017). Even symptoms of PTSD patients could be improved by interfering with reconsolidation via the application of propranolol after retrieval (Brunet and Monson, 2014; Kindt and van Emmerik, 2016). Furthermore, human reconsolidation has been reported not only in procedural or aversive memories but also in declarative memories (Hupbach et al., 2007; Chan and LaPaglia, 2013; Sinclair and Barense, 2018).

However, it remains controversial as to whether reconsolidation can be generally applied to every type of memory because there are also reports showing no effect of interfering with reconsolidation on memory performance (Golkar et al., 2012; Hardwicke et al., 2016). This debate appears to be especially more pronounced in the two conditions; declarative memory and old memory. Here, we focus on controversial results associated with the reconsolidation of declarative memory and old memory in human subjects and suggest that the controversial findings may be mainly related to the complexity and range of the cortical circuits involved in a particular memory.

## Reconsolidation of Human Declarative Memory

To apply reconsolidation theory to the area of human memory modification, including its application as a clinical treatment for PTSD, it is necessary for both declarative and non-declarative components of traumatic memory to be susceptible to reconsolidation. However, while most reconsolidation studies show consistent results supporting the contention that implicit fear memory undergoes post-retrieval reconsolidation, it remains controversial as to whether reconsolidation can be applied to declarative memory types, including declarative fear memory (Schiller and Phelps, 2011; Soeter and Kindt, 2015). Soeter and Kindt (2015) showed that interference with the systematic administration of propranolol after retrieval induced the erasure of startle fear but did not affect unconditioned stimulus (US) expectancy ratings. Björkstrand et al. (2016) demonstrated that the disruption of reconsolidation could facilitate an approach for individuals with a lifelong fear of spiders to new spider stimuli, and Xue et al. (2012) showed that extinction training 10 min after retrieval could effectively attenuate cue-induced heroin cravings, but these studies did not examine declarative memory components separately. Moreover, there was little effect of interference through a reconsolidation process for disrupting the restabilization of traumatic naturalistic memories in humans (Shiban et al., 2015; Maples-Keller et al., 2017; Telch et al., 2017).

Several studies have investigated the reconsolidation of neutral declarative memories based on tasks using lists of words or objects. In these tasks, participants were asked to memorize a word or object list and to learn a second list immediately after the retrieval of the first list (Hupbach et al., 2007, 2009; Dongaonkar et al., 2013; Hupbach, 2015). In subsequent retrieval tests, the participants showed impairment of the original list memory, while the control group who underwent the same procedure except for retrieval just before the second learning showed maintenance of the original list (Hupbach et al., 2007; Dongaonkar et al., 2013; Hupbach, 2015). Additionally, many studies have reported that relearning after the reactivation of the original memory can strengthen memory in both young and old adults, suggesting a memory enhancement through reconsolidation (Forcato et al., 2011, 2013; Tassone et al., 2020). These results suggest that reconsolidation commonly occurs in human declarative memories. However, there are also compelling research results that raise the question of whether the reconsolidation process can be generally applied to declarative memory. In similar tasks based on list learning, the second learning after memory retrieval had no effect on the memory of the original list (Hardwicke et al., 2016; Klingmüller et al., 2017).

These controversial results have also been reported for more naturalistic event memories. Chan and LaPaglia (2013) demonstrated that misinformation following the reactivation of an original memory of a movie clip can interfere with reconsolidation, leading to the impairment of the original episodic memory. Moreover, James et al. (2015) used computer gameplay as a reconsolidation interference tool, showing that traumatic memories were successfully reduced. Sinclair and Barense (2018) also reported that simply watching mismatching movie clips after the reactivation of the original episodic memory can disturb the reconsolidation process, influencing subsequent episodic memory performance outcomes. Additionally, reminders to reactive film memories recovered and prevented a loss of detailed episodic memory (Sekeres et al., 2016), suggesting the strengthening of memory through a reconsolidation process. Moreover, in work by St. Jacques and Schacter (2013) based on a museum tour paradigm, retrieval was shown to enable memories to be both selectively enhanced and distorted via updating. However, there are also inconsistent findings on episodic memory (Rindal et al., 2016; Elsey et al., 2018). Specifically, Rindal et al. (2016) failed to replicate the findings of Chan and LaPaglia (2013). Unlike Chan and LaPaglia (2013), they measured hit and false alarm rates separately. As a result, they found that there was no significant increase in false alarm rate when misinformation was given with retrieval, suggesting no evidence of memory impairment by the integration of misinformation after retrieval. Schwabe and Wolf (2009) showed that while new episodic learning interfered with the reconsolidation of neutral autobiographical memories, the same paradigm was not effective for emotional memories.

With regard to why a substantial number of studies observed a failure of memory reconsolidation, many studies indicate boundary conditions on the induction of reconsolidation (Nader and Einarsson, 2010; Walker and Stickgold, 2016; Elsey and Kindt, 2017; Lee et al., 2017; Monfils and Holmes, 2018). They suggest that stronger memories, usually resulting from repetitive training or exposure to highly attentional conditions such as stressful situations, or older memories are relatively less susceptible to reconsolidation effects (Milekic and Alberini, 2002; Wang et al., 2009; Bustos et al., 2010; Elsey and Kindt, 2017). However, the proposed boundary conditions do not seem to be absolute. Strong fear memories, which are resistant to extinction, were still susceptible to the disruptive effects of propranolol given with memory retrieval (Soeter and Kindt, 2012). Moreover, Fernández et al. (2016a) also showed that strong declarative memory acquired under stress or old memory could still be destabilized and impaired based on a reconsolidation procedure, suggesting that the boundary conditions of reconsolidation are not fixed but depend on memory features and the reminder characteristics.

There is also speculation suggesting that memory reconsolidation depends on the arousal level during the memory processes. These studies claim that the effect of the reconsolidation blockade is limited to specific experimental conditions with a high arousal level, such as in fear-conditioning memory (Lewis, 1976; Squire et al., 1976; Fernández et al., 2016b). However, given the findings related to reconsolidation in non-emotional semantic and episodic memories (Hupbach et al., 2007; Chan and LaPaglia, 2013), a high arousal level may not be indispensable for triggering the reconsolidation process. Other studies also suggest that prediction errors or novel information during memory retrieval would be critical for triggering reconsolidation. It was found that behavioral interference or a protein synthesis block after memory retrieval did not affect the original memory when no new information was given (Sinclair and Barense, 2019). However, it appears that prediction error is necessary for the reconsolidation process but is not sufficient to induce reconsolidation (Sevenster et al., 2014). Therefore, it is necessary to gain a better understanding of the more fundamental neural processes that determine the boundary conditions.

## Effect of Age of Memory

Another controversial issue related to the general application of reconsolidation is whether reconsolidation can be triggered regardless of how old the memory is. Despite numerous studies suggest the possibility that reconsolidation can be used to modify long-term memories, it has also been reported that older memory is less susceptible to reconsolidation. Thus, as mentioned above, the age of memory has been considered as one of the major boundary conditions of the induction of reconsolidation. Prior animal studies showed that while pharmacological interference after retrieval impaired recent memory (1 day or 1 week), remote memory (more than 2 weeks or 1 month) was less influenced (Milekic and Alberini, 2002; Eisenberg and Dudai, 2004; Suzuki et al., 2004; Frankland et al., 2006). Some studies also suggest that this difference may reflect the gradual decrease of hippocampal involvement with increasing memory age (Dudai, 2004; McKenzie and Eichenbaum, 2011; Squire et al., 2015). However, there is also evidence indicating that remote memories undergo reconsolidation after retrieval, suggesting ACC as neural substrates underlying the reconsolidation of remote memory (Frankland et al., 2004; Einarsson and Nader, 2012). Reengagement of the hippocampus in remote memory reconsolidation was also considered (Debiec et al., 2002; Myers and Davis, 2002).

The dependency of memory reconsolidation on the age of the memories has also been examined in human research. Wichert et al. (2011) compared memory impairment of 1-, 7-, and 28-day-old memories after reactivation followed by behavioral interference, finding that 1- and 7-day-old memories but not 28-day-old memories were impaired by post-reactivation interference. Steinfurth et al. (2014) suggested no effect of memory age, but they examined only 1 and 7-day-old memories based on the Pavlovian fear memory paradigm. However, it is hasty to conclude that older memories are less susceptible to reconsolidation because there is also evidence supporting the modification of human remote memories (Cohen and Squire, 1981).

## Involvement of Cortical Circuits in Reconsolidation

To utilize reconsolidation as a tool to modify human memory, including as a clinical treatment, reconsolidation must be a general property of memories. However, as reviewed above, controversy remains in particular with regard to whether declarative memory undergoes reconsolidation and whether even old memories can be reorganized after retrieval. We suggest the possibility that these two issues are ultimately related to how different and complex cortical circuits are involved in a given memory.

While implicit fear memory induced by Pavlovian conditioning involves relatively simple neural circuits centered at the amygdala, declarative memories appear to involve broadly distributed neural circuits across different brain regions. The medial temporal lobe (MTL) regions, including the hippocampus, are well-known as a key system for the encoding, consolidation and retrieval of declarative memories (Gluck and Myers, 1997; Tulving and Markowitsch, 1998; Teyler and Rudy, 2007; Moscovitch et al., 2016). Not only the MTL regions but also diverse cortical regions are involved in declarative memory processing. Prior studies suggest that specific types of sensory information are stored in the sensory cortical regions that are specialized for the processing of those types of information (Gandhi, 2001; Hofstetter et al., 2012; Lee et al., 2019). The association cortex is also involved in linking identical or different modality information in declarative memory processes (Svoboda et al., 2006; Cabeza and St Jacques, 2007; Rugg and Vilberg, 2013). Furthermore, the frontal cortical regions are considered to be engaged in the processing of higher-level conceptual information and executive control of memory processes (Tomita et al., 1999; Badre and Wagner, 2002; Preston and Eichenbaum, 2013). Specifically, Sekeres et al., showed that memory retrieval activates the ventrolateral prefrontal cortex (vlPFC) followed by the hippocampus (Sekeres et al., 2021).

Although episodic and semantic memories are inextricably intertwined and show considerable overlap in their neural substrates, they nonetheless retain a measure of distinctiveness (Renoult et al., 2019). In particular, while the detailed contextual information of episodic memory is considered to involve the hippocampus (Vargha-Khadem et al., 1997; Eldridge et al., 2000; Eichenbaum et al., 2007), gist information or semantic memory is thought mainly to depend on the cortical regions (Patterson et al., 2007; Binder and Desai, 2011; Moscovitch et al., 2016). Thus, even within declarative memory, the extent to which cortical circuits are involved may depend on the contents of the memory. The time-dependent loss of the peripheral details of episodic memories compared to the lower levels of the loss of the gist of events may reflect this different degree of involvement of the cortical and hippocampal circuits (Sekeres et al., 2016).

The involvement of diverse and complex cortical circuits may be more prominent in older memories. Memory models suggest that locally consolidated memory traces undergo system-level consolidation, which involves the reorganization of memory traces across different neural circuits and regions (Dudai, 2004; Barry and Maguire, 2019). This system-level consolidation process occurs over weeks, months, or even years after new information is learned, while synaptic consolidation is thought to occur within hours or days (Dudai, 2004). According to standard consolidation theory, declarative memory is initially encoded in a hippocampal-cortical trace but, over time, is stored in progressively strengthened cortico-cortical connections while the hippocampal trace fades (Squire et al., 2015). Multiple trace theory or recent scene construction theory also suggests the progressive development of a cortico-cortical trace over time but argues a perpetual role of the hippocampus in the retrieval of memory representations (Nadel et al., 2007; Moscovitch et al., 2016; Barry and Maguire, 2019). Although these views on system-level consolidation suggest different roles of the hippocampus in memory consolidation, they all suggest that stabilization of a memory progressively engages more cortico-cortical connections over time. Therefore, more cortical circuits may be involved in older memories, even for simple types of memory.

The fact that memory involves more diverse and broadly distributed cortical circuits indicates that it is more difficult to modify the memory via a reconsolidation process in two aspects. First, due to the diversity and complexity of neural circuits, there is a possibility that interference signals after memory retrieval may not sufficiently reach the core circuits underlying the target memory trace. If only partial circuits are affected, it is possible that other unaffected circuits compensate for the interfering effect. Of course, whether interfering partial circuits are effective may depend on how much the core circuits are affected. Therefore, it will be important to clarify how we can effectively interfere with the core circuits underlying the target memory in future reconsolidation research. The experiments with non-invasive brain stimulation methods such as transcranial magnetic stimulation (TMS) or direct current stimulation (tDCS) can contribute to uncover the core circuits in humans. Recent brain stimulation studies have shown that stimulating or disturbing the neural activity of a specific region after memory retrieval can modify the target episodic memory (Table 1). For example, anodal transcranial direct current stimulation (tDCS) on the lateral prefrontal cortex (lPFC) after memory reactivation enhanced episodic memory recognition in healthy young and old adults (Javadi and Cheng, 2013; Sandrini et al., 2014) as well as old adults with subjective memory complaints (SMC) (Manenti et al., 2017) or amnestic mild cognitive impairment (aMCI) (Manenti et al., 2020). Moreover, repeated TMS to the medial PFC of PTSD patients after brief exposure to a traumatic event with an imagery procedure provided a significant therapeutic effect in these patients (Isserles et al., 2013). On the other hand, over the left posterior parietal cortex, no effect of tDCS during the reconsolidation of word memory was reported (Crossman et al., 2019). Based on these results, we can consider the prefrontal cortex as a candidate region that includes the core circuits of declarative memory processes.

**TABLE 1:** Recent human reconsolidation studies of declarative memory using brain stimulation as an intervention tool.

Authors	Paradigm	Population (age)	Intervention	Target region	Effect (Yes/No)
Javadi and Cheng (2013)	Word list learning	Healthy young adults (mean age 22.6 years, range 18–24 years)	Anodal tDCS at 1.5 mA	Left dlPFC	Application of anodal tDCS with memory reactivation resulted in memory enhancement at 5 h after retrieval. (Yes).
Sandrini et al. (2013)	Word list learning	Healthy adults (mean age 24.9 years, range 23–30 years)	1 Hz rTMS	Right lPFC	Application of rTMS after memory reactivation strengthened memories at 1-day but not 1 h after retrieval (Yes).
Kroes et al. (2014)	Emotional episodic memory recall	Adults with unipolar depression (mean age 57.2 ± 4.0 years)	ECT	Right unilateral or bifrontal temporal area	ECT application following memory reactivation in unipolar-depressed patients disrupted reactivated emotional episodic memories (Yes).
Sandrini et al. (2014)	Word list learning	Healthy old adults (mean age 67.2 ± 3.7 years)	Anodal tDCS at 1.5 mA	Left DLPFC	Application of anodal tDCS after a contextual reminder strengthened existing memories and reduced forgetting in healthy older subjects compared to the sham stimulation with reactivation group (Yes). A sham-no reminder group was not tested.
Manenti et al. (2017)	Word list learning	Old adults with SMC (mean age 74.5 ± 5.9 years)	Anodal tDCS at 1.5 mA	Left lateral PFC	Application of anodal tDCS after a contextual reminder strengthened existing memories up to 30 days, compared to sham stimulation (Yes).
Manenti et al. (2020)	Word list learning	Old adults with aMCI (mean age 75.3 ± 3.7 years)	Anodal tDCS at 1.5 mA	Left lateral PFC	Application of anodal tDCS after contextual reminder strengthened existing memories up to 30 days, compared to sham stimulation (Yes).
Crossman et al. (2019)	Word list learning	Healthy young adults (mean age 20.9 ± 2.9 years)	Anodal tDCS at 1.5 mA	Left ventral PPC	No significant differences in the mean number of words recalled on day 3 were found between anodal tDCS with reactivation group, anodal tDCS without reactivation group, and anodal tDCS over primary visual cortex group (No).

*SMC, subjective memory complaints; aMCI, amnestic mild cognitive impairment.*

The second aspect of difficulty when attempting to modify memory via a reconsolidation process can arise from the property of cortical synapses. Because cortical synapses are known to have relatively slow plasticity compared to the property of the hippocampal synapses (Nakazawa et al., 2003; Dudai, 2004; Horner and Doeller, 2017), reconsolidation of a memory trace that involves more cortical circuits may need stronger and longer-lasting interfering signals after retrieval.

Thus, the controversial results on the reconsolidation of declarative memory or old memory may be due to the different degrees of the involvement of the cortical circuits and the different intensity levels of the interfering signals affecting them. Given that there are reports showing that reconsolidation can be triggered by interfering with or facilitating the activity of particular cortical circuits (Javadi and Cheng, 2013; Sandrini et al., 2013; Censor et al., 2014; Manenti et al., 2017; Borgomaneri et al., 2020) and that the same synapse that undergoes simple memory consolidation can be also disrupted and reconstructed after retrieval (Lee et al., 2012), it is still hopeful that reconsolidation is a general property of memory. If interfering signals with sufficient intensity levels and ranges are given, even declarative memories or old memories may be reconsolidated or modified. Further studies need to be done to elucidate such conditions for the induction of reconsolidation of consolidated memories. In line with that, it will be important to uncover neural circuits that are core to any type of memory trace and to reveal how the hippocampal circuits, which show the property of fast plasticity, interact with cortical circuits. These studies may critically contribute to the formulation and general application of reconsolidation in the development of effective interventions as part of a treatment regime for mental disorders related to maladaptive memories.

## Author Contributions

GK, MK, WK, and S-HL wrote the manuscript. GK and S-HL conceptualized the manuscript. S-HL supervised and edited the manuscript. All authors contributed to the article and approved the submitted version.

## Conflict of Interest

The authors declare that the research was conducted in the absence of any commercial or financial relationships that could be construed as a potential conflict of interest.
